# Draft Genome Sequence of *Amantichitinum ursilacus* IGB-41, a New Chitin-Degrading Bacterium

**DOI:** 10.1128/genomeA.01309-15

**Published:** 2015-11-19

**Authors:** Philipp Kirstahler, Michael Günther, Christian Grumaz, Elena Lindemann, Steffen Rupp, Susanne Zibek, Kai Sohn

**Affiliations:** Fraunhofer IGB, Stuttgart, Germany

## Abstract

*Amantichitinum ursilacus* IGB-41 is a new species of chitin-degrading bacterium isolated from soil, which secretes potential industrial enzymes. The genome of *A. ursilacus* was sequenced, and the gene set encoding chitinases was identified. Here, we present the draft genome of 4.9 Mb, comprising 38 contigs, and the corresponding annotation.

## GENOME ANNOUNCEMENT

Chitin is the second most abundant biopolymer on earth and can be found as a structural polysaccharide in a variety of organisms, including fungi, crustaceans, and insects (1). The polymer consists of *N*-acetylglucosamine and glucosamine, two amino sugars that are of increasing economic interest, e.g., as food additives, cosmetic ingredients, and platform chemicals (2, 3). Compared to cellulose, chitin is much more resistant to biodegradation, and only the concerted action of different classes of chitinases is able to cleave the molecule efficiently (4). Due to the absence of commercially available chitinase cocktails, we screened for microorganisms with abundant chitinolytic activity. The most promising microbial isolate was a new species we found, which was named *Amantichitinum ursilacus* IGB-41 (5). The strain secretes an enzyme cocktail able to hydrolyze crab chitin into its monomers.

Here, we describe the draft genome sequence of *A. ursilacus* IGB-41. For this purpose, extracted DNA was prepared on one hand for Illumina HiSeq 2000 with the TruSeq DNA kit and on the other hand for Roche GS Junior with the Rapid Library/emPCR Lib-L kit, in both cases using standard protocols. A total of 3,161,184 reads with a length of 70 bp by Illumina sequencing, and 147,252 reads with a mean length of 458 bp from 454 sequencing, were generated, resulting in average genome coverages of 44× and 13×, respectively. The Illumina reads were removed for contaminations and adapter sequences with BBDuk from the BBMap package version 34.41 (http://sourceforge.net/projects/bbmap/). The genome was assembled using GS *de novo* Assembler (version 2.9), generating 38 contigs with an *N*_50_ of 323 kb. The average contig length in the presented genome is 129 kb.

The assembled sequences had a total of 4.9 Mb, with a G+C content of 59.63%. Gene annotation was carried out using Prokka 1.11 (6), providing 4,423 potential open reading frames (ORFs). To unravel the genetic background of this chitinolytic machinery, we searched for characteristic enzyme domains of chitinolytic hydrolases, revealing a set of four endochitinases and one *N*-acetylglucosaminidase. The *in silico* search for conserved enzyme domains resulted in a set of 10 genes: 8 endochitinases of the GH18 family responsible for cleavage inside the polymer chain, one *N*-acetylhexosaminidase of the GH20 family, which catalyzes cleavage of the dimer chitobiose to *N*-acetylglucosamine, and one chitinase AN-terminal domain potentially involved in an interaction with chitin. The obtained gene sequences can now recombinantly be expressed as hydrolytic enzymes with promising potential.

### Nucleotide sequence accession numbers.

This whole-genome shotgun project has been deposited at DDBJ/EMBL/GenBank under the accession no. LAQT00000000. The version described in this paper is version LAQT01000000.
